# Anti-PD-1 Immunotherapy and Bee Venom for Relapsed and Refractory Liposarcoma: A Case Report

**DOI:** 10.3389/fonc.2021.668992

**Published:** 2021-04-29

**Authors:** Wei Yang, Yeke Zhang, Gaoyi Yang, Yanhua Geng, Da Chen, Jun Wang, Yang Ye, Huaichong Wang, Dajing Xia, Fuliang Hu, Jing Jiang, Xiaofeng Xu

**Affiliations:** ^1^ Department of Oncology, Hangzhou Red Cross Hospital, Hangzhou, China; ^2^ Department of Medical Imaging, Hangzhou Red Cross Hospital, Hangzhou, China; ^3^ Department of Pathology, Hangzhou Red Cross Hospital, Hangzhou, China; ^4^ Department of Thoracic Surgery, Hangzhou Red Cross Hospital, Hangzhou, China; ^5^ Department of Traditional Chinese Medicine, Hangzhou Red Cross Hospital, Hangzhou, China; ^6^ Department of Pharmacy, Hangzhou Red Cross Hospital, Hangzhou, China; ^7^ Department of Toxicology of School of Public Health, Zhejiang University School of Medicine, Hangzhou, China; ^8^ College of Animal Sciences, Zhejiang University, Hangzhou, China

**Keywords:** bee venom, PD-1 inhibitor, STING pathway, immunotherapy, apitherapy, liposarcoma, innate immunity, complementary medicine

## Abstract

Cancer immunotherapies, including immune checkpoint inhibitors, elicit long-term clinical responses but many cancer patients do not respond. Intensive efforts are therefore underway to identify additional immune pathways that may be modulated to enhance the efficacy of existing immunotherapies. Bee venom strongly stimulates the immune system, and is used as a complementary therapy to treat cancer pain in patients with advanced tumors in China. Bee venom contains several allergenic protease inhibitors and peptides. It triggers hypersensitivity reactions; that is, it is an immune system agonist. The generation of a spontaneous T cell response against tumor-associated antigens requires innate immune activation; this drives type I interferon production. We report a patient with a relapsed and refractory liposarcoma who had undergone several operations, chemotherapies, and radiotherapies. The tumor was large. The patient had attained the maximum radiation exposure dose. The tumor was resistant to chemotherapy and was infiltrating the pericardium, lungs, and diaphragm. The patient was a poor candidate for resection. He thus received apitherapy (a combination of bee venom and acupuncture) to control pain; then apatinib (an anti-angiogenic drug) was given to inhibit tumor growth but was terminated early because the patient could not tolerate the side effects. Subsequently, a programmed death 1 inhibitor was combined with apitherapy. Bee venom served as an innate immune system agonist promoting immune cell priming and recruitment in the tumor microenvironment. The patient was finally able to undergo radical liposarcoma resection, and no evidence of recurrence was found at re-examination 16 months after surgery.

## Introduction

Apitherapy, a therapy unique to traditional Chinese medicine, features both acupuncture and moxibustion (the burning and swelling caused by bee venom is similar to moxibustion). Acupuncture reduces cancer pain (1). This analgesic effect is enhanced by bee venom; so apitherapy (a complementary medicine) has often been used to relieve pain (2). In our hospital, we use live honeybees to sting patients with terminal cancer; this ensures venom purity. We use thin-tipped forceps to hold the waist of the honeybee and press the sterilized tail of the bee against the patient’s skin. All venom in the sac becomes subcutaneously injected after 20 min, and then the bee needle is removed using a blunt forceps. Patients with cancer pain who refuse opioids or for whom opioids are not effective, and who require additional adjuvant therapy, are offered apitherapy after a full explanation and the obtaining of written informed consent.

Drugs that target programmed death 1 or programmed death 1 ligand 1 (PD-1/PD-L1) (immune checkpoint inhibitors) have become widely available in recent years (3), but few patients exhibit objective responses when given these materials alone (4). Activation of the stimulator of interferon genes (STING)-controlled innate immune pathway is a promising therapeutic strategy that induces tumor regression with long-term antitumor immunity. Such activation is synergistic with the effects of PD-1/PD-L1 inhibitors (5). A bee sting (venom) is a possible STING agonist. Thus, we combined a PD-1 inhibitor and apitherapy to treat a patient admitted to our oncology department in 2019.

## Case Presentation

**Figure 1:**
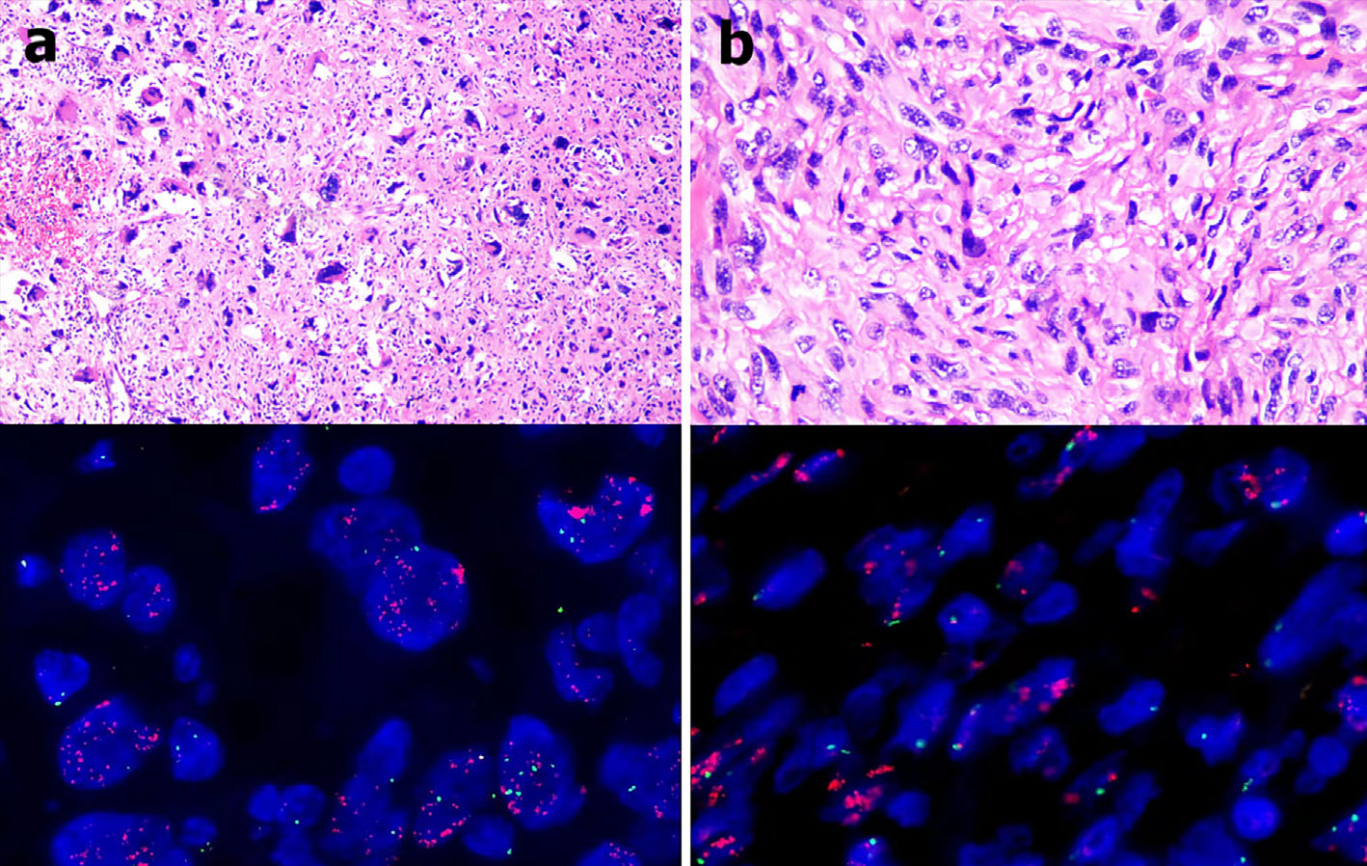
**(A)** The 2014 pathological examination indicated a pleomorphic liposarcoma (hematoxylin and eosin staining, 400 × magnification) and MDM2 amplification in tumor tissues as revealed by fluorescence *in situ* hybridization (red fluorescence: MDM2, green fluorescence: CEP12; mean MDM2 signal: 35.1, mean CEP12 signal: 1.7, MDM2/CEP12 ratio = 20.65). **(B)** The 2017 pathological examination indicated a de-differentiated liposarcoma (hematoxylin and eosin staining, 400 × magnification) and MDM2 amplification in tumor tissues as revealed by fluorescence *in situ* hybridization (red fluorescence: MDM2, green fluorescence: CEP12; mean MDM2 signal: 27.4, mean CEP12 signal: 1.8, MDM2/CEP12 ratio = 15.22).

The patient was a 62-year-old man with a liposarcoma that had repeatedly relapsed. In February 2000, he underwent resection of a lipoma-like nodule on the left wall of the chest, but surgical pathology was not performed. In June 2004, he found a lump on the edge of the surgical scar. Three months later, a tumor was removed in the same hospital but again no tissue was sent for pathological examination. One month later, another mass developed on the left wall of the chest, and grew rapidly to attain dimensions of about 5 × 4 cm. He was admitted to a general hospital and underwent locally expanded, left wall excision on March 28, 2005. The surgical pathology indicated a pleomorphic liposarcoma with positive surgical margins. Then the patient underwent nine rounds of chemotherapy (combined pirarubicin, vindesine, and cyclophosphamide for the first three rounds; combined pirarubicin and dacarbazine for the second three rounds; and combined epirubicin and ifosfamide for the last three rounds). Radiotherapy (30 Gy) was delivered to the surgical area of the left wall of the chest after chemotherapy concluded. The liposarcoma recurred once more (on October 30 2014) 5 cm below the left nipple. Four weeks later, he underwent a repeat, locally expanded excision of the same wall. A pleomorphic liposarcoma with positive surgical margins was again confirmed by surgical pathology (**Figure 1A**). After two more rounds of chemotherapy (combined epirubicin and ifosfamide), he received a further 50 Gy of radiotherapy to the site. However, the incision cracked and did not heal. After a series of surgeries including expanded debridement, soft tissue repair using a latissimus dorsi myocutaneous flap, and skin grafting, the wound gradually healed. He detected a 1 × 2 cm painless scleroma above the scar on December 2, 2017. Three weeks later, he again underwent locally expanded excision at the same site. The fifth rib had been invaded by the tumor and was partially removed during operation. A de-differentiated liposarcoma with a negative surgical margin was confirmed by surgical pathology (**Figure 1B**).

**Figure 2:**
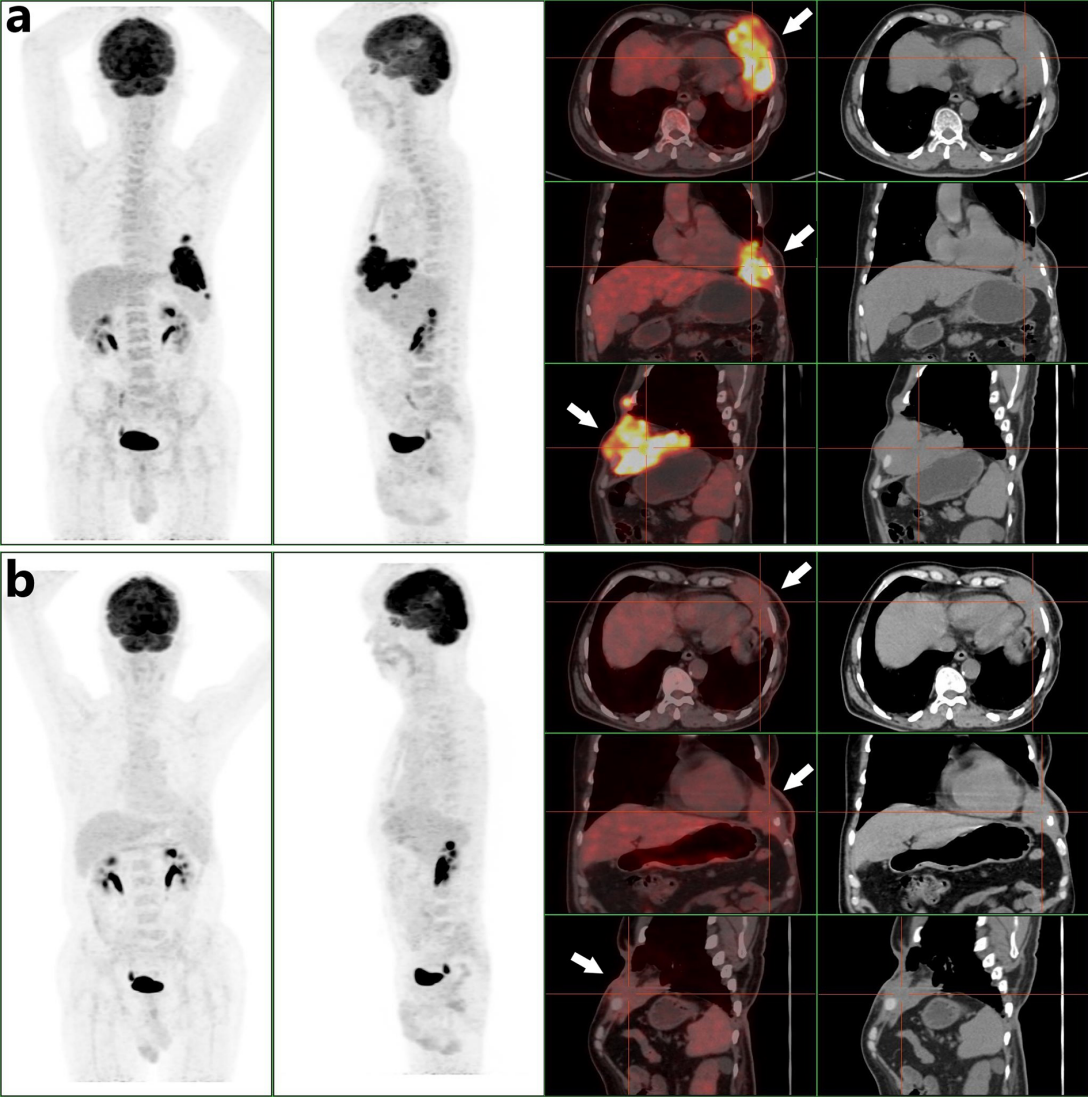
**(A)** A PET/CT scan obtained before combined oncotherapy on April 9 2019 (white arrows, the tumor mass with high FDG metabolism). **(B)** A PET/CT scan obtained before surgery on September 30 2019 (white arrows, the tumor mass was smaller than before and its FDG metabolism was less than before).

On February 7, 2019, he experienced local pain and swelling on the left wall of the chest. The pain commenced as a pinprick, but then developed into a paroxysmal tear-like shape with a numerical rating scale (NRS) score of 6. The pain was not relieved by loxoprofen sodium tablets, and his sleep was severely affected. On April 8 2019 the patient was admitted to our oncology department for the first time. Positron emission tomography (PET)/computed tomography was performed early the following day and revealed a huge mass with irregular nodules and protrusions, of dimensions 10.5 × 5.4 × 7.2 cm, in the lower left wall of the chest. The boundary of the mass was unclear, and it was lobulated. The density was uneven, and local fat density shadows were evident. The average mass density was about 49.5 Hounsfield units. The mass exhibited enhanced fluorodeoxyglucose (FDG) metabolism, with an SUVmax of 15.07. The mass had invaded part of the pericardium, diaphragm, and interior of the left lung. The left fifth anterior rib was absent, and the cortex of the left sixth anterior rib was irregular. The FDG metabolism of the left sixth anterior rib was elevated, with an SUVmax of 3.2 (**Figure 2A**). The radiologist concluded that the patient could not receive any more radiotherapy. The thoracic surgeon considered that as the tumor was so large, and had invaded the pericardium, diaphragm, and lung tissue, the surgical risk was high and it would be difficult to ensure a negative surgical margin. Thus, surgery was not recommended at that time. On the same afternoon, we commenced apitherapy at the bulge of the left sixth rib (we used the bee *Apis cerana fabricius*).

**Figure 3:**
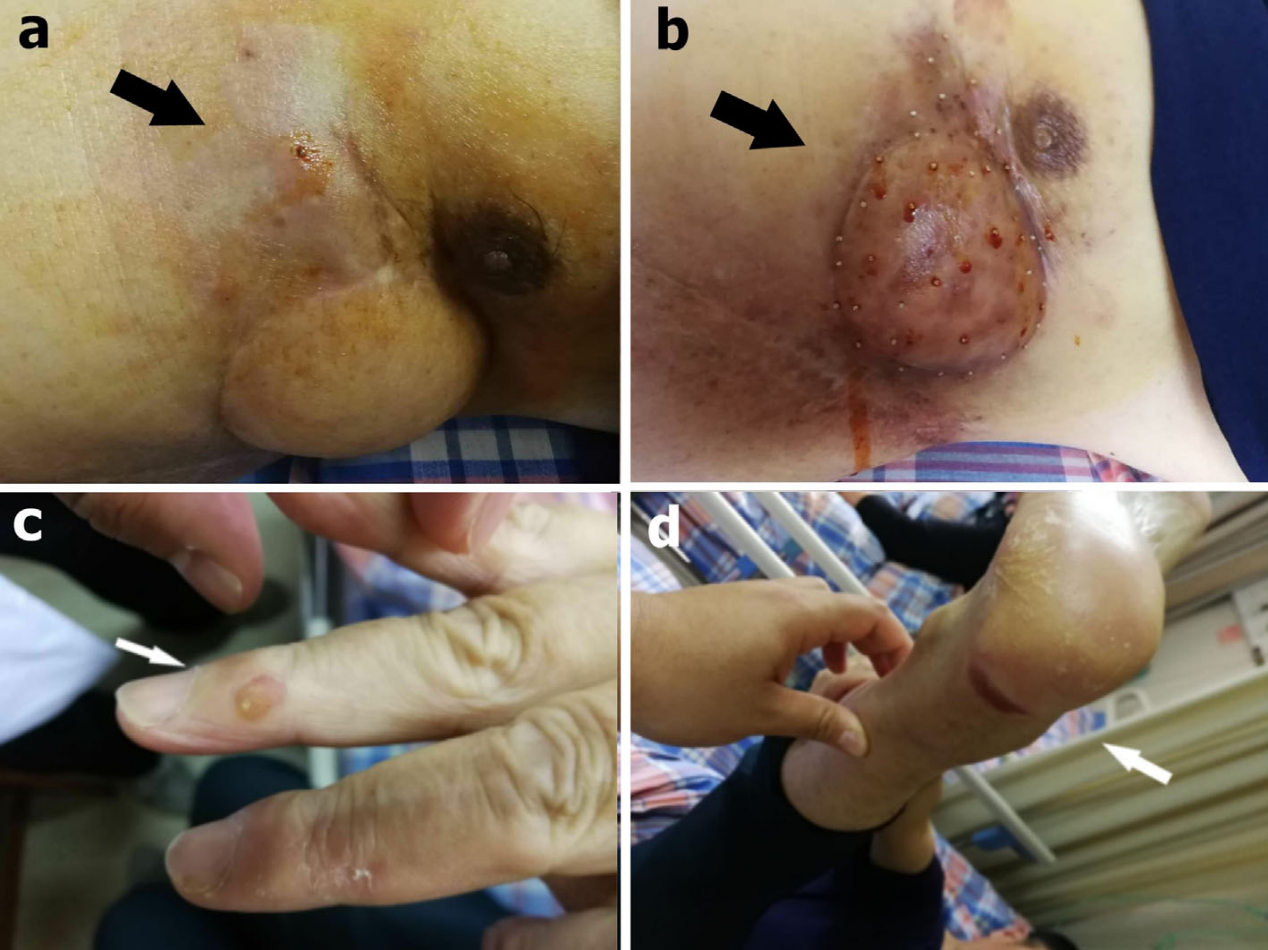
**(A)** During the first apitherapy session, the patient was stung by only one bee and observed in a ward for 2 h (black arrow). **(B)** After 9 weeks, the dose had gradually increased to 50 bee stings each time (black arrow). **(C)** A finger ulcer (white arrow) and **(D)** a heel ulcer were side effects of apatinib (white arrow).

We initially used only one bee to sting (**Figure 3A**). The NRS score dropped to 1 and the patient slept throughout the night. Then he underwent apitherapy on Mondays, Wednesdays, and Fridays; the dose was gradually increased to 18 bee stings each time over 6 weeks. The thoracalgia that had interfered with his sleep did not recur. Apitherapy focused principally on the chest wall area corresponding to the tumor mass indicated by PET/CT (**Figure 2A**). He commenced apatinib (Jiangsu Hengrui Medicine Co. Ltd.) on April 12 2019 (425 mg once daily). Apatinib is a specific molecular inhibitor of VEGFR-2 and exhibits antitumor activity both *in vivo* and *in vitro* (6, 7). We discontinued it on the weekends because the patient developed painful ulcers of the fingertips, heels, and throat after 4 weeks on the drug (**Figures 3C, D**). After another 2 weeks, we reduced apatinib to three times weekly because he complained of a constant sore throat. We increased the apitherapy dose from 18 to 50 bee stings each time, and discontinued apatinib because the sore throat and consequent dysphagia did not resolve within 3 weeks (**Figure 3B**). The sore throat and limb extremity ulcers disappeared after apatinib withdrawal. He was given intravenous infusions of camrelizumab (Jiangsu Hengrui Medicine Co. Ltd.), a PD-1 inhibitor that recently received conditional approval in China for treatment of relapsed or refractory classic Hodgkin lymphoma, non-small cell lung cancer, hepatocellular carcinoma, and esophageal squamous cell carcinoma. He received the drug on June 19, July 10, July 31, August 21, and September 1 2019 (200 mg every 3 weeks). Apitherapy was discontinued on September 20 2019. Ten days later, he underwent repeat PET/CT (**Figure 2B**) and then a complex surgery featuring enlarged resection of the chest wall tumor, thoracoplasty, and surgical repair of the chest wall, pulmonary, and diaphragm defects.

**Figure 4:**
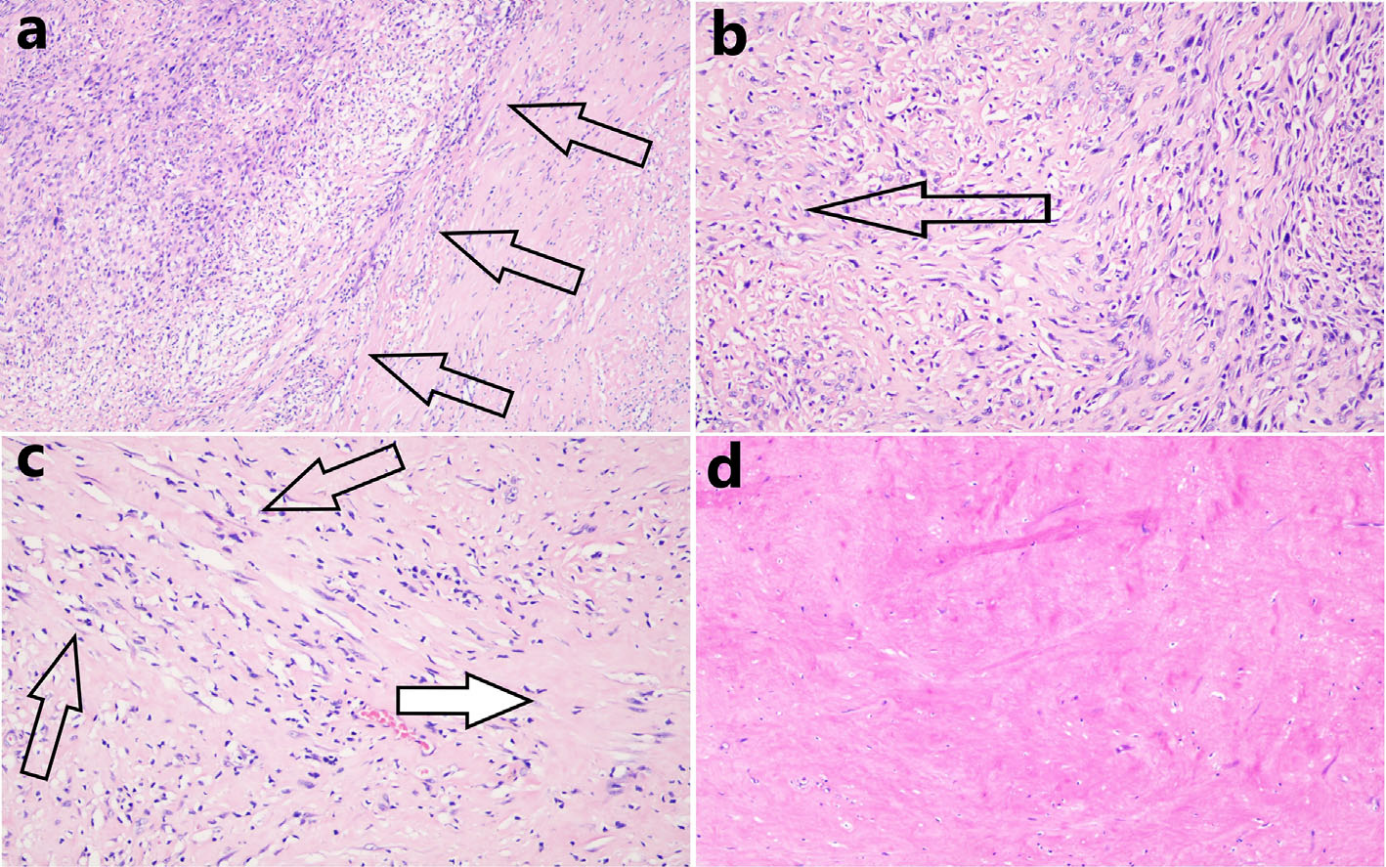
**(A)** In the section with most residual tumor tissue, the tissue area was about 0.3 × 0.2 cm and the boundary (black arrows) between that tissue and the hyaline degeneration was distinct (hematoxylin and eosin staining, 100 × magnification). **(B)** Many tumor cells were observed near remnant tumor tissue, but these gradually decreased in number in the distal areas (the direction shown by the black arrow, hematoxylin and eosin staining, 200 × magnification). **(C)** The closer the transition zone of hyaline degeneration, the thinner the density of tumor cells (black arrows, residual tumor cells), and the greater the extent of necrotic degeneration (white arrow, hematoxylin and eosin staining, 200 × magnification). **(D)** Complete hyaline degeneration (without residual tumor cells) was observed in all other sections (hematoxylin and eosin staining, 200 × magnification).

We completely removed the tumor along the outer margin of the mass. The mass dimensions were now 8 × 5 × 3 cm. Pathology showed that the mass exhibited a degenerative change characterized by hyaline degeneration of sections of the surgical specimens. Hemangiogenesis and chronic inflammatory cell infiltration were evident at the edge of the mass, but no tumor cells were found in the ribs or vessels or at the excisional margins of the pericardium, lungs, or diaphragm. Of all sections, only 41 consecutive sections exhibited residual tumor tissue. In the section with most such tissue, the tumor area was about 0.3 × 0.2 cm (**Figures 4A–D**).

**Figure 5:**
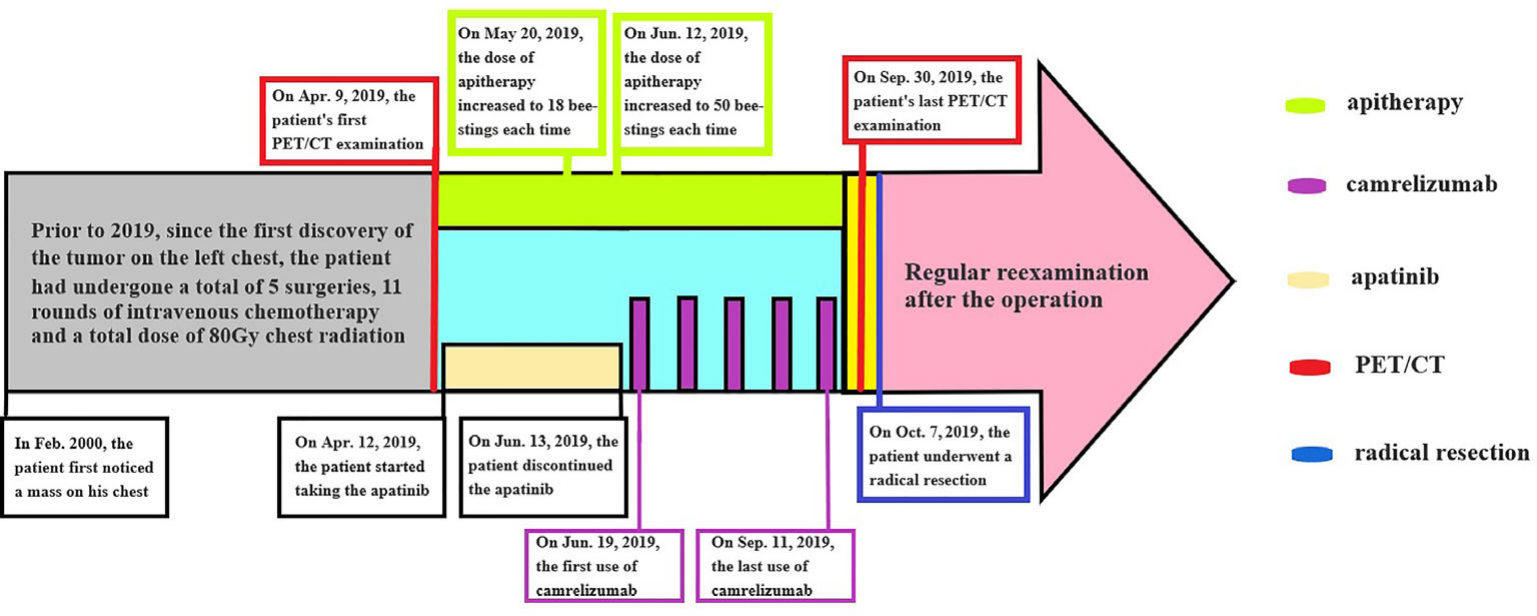
A flowchart/timeline of the therapy.

Prior to the radical resection of October 7 2019, the patient had been battling his recurrent liposarcoma for 20 years, and had undergone many surgeries, chemotherapies, and radiation treatments. He was physically and mentally exhausted. At his request, we discontinued all antitumor therapy after surgery. The patient receives a medical re-examination every 4 months, and no evidence of tumor recurrence was apparent to his fourth follow-up (thus to 16 months after surgery) (**Figure 5**).

## Discussion

The surgical pathologies confirmed a histological transformation from a pleomorphic liposarcoma to a de-differentiated liposarcoma, but murine double-minute 2 gene (MDM2) amplification in tumor cells was detected by fluorescence *in situ* hybridization at all times (**Figure 1**). MDM2 encourages cancer progression; it negatively regulates p53 gene activity, being part of an autoregulatory feedback loop that controls the levels of both proteins (8). MDM2 expansion is supposed to indicate hyperprogression of a malignant tumor after immunotherapy. Thus, we preferred apatinib to camrelizumab (9, 10). However, anti-tumor therapy using apatinib alone may trigger drug-resistance, particularly if the apatinib dose is reduced (11). Immune checkpoint inhibitors are used to treat soft tissue sarcomas (12, 13). Some clinical studies have suggested that the combined use of apatinib and a PD-1 inhibitor imparts a synergistic effect (14, 15). We initially planned to add a PD-1 inhibitor (apatinib). However, the patient could not tolerate the side effects; we discontinued apatinib on June 13 2019. As the giant wound on the left chest remained open after the second course of radiotherapy, the radiologist emphasized that a third course of radiotherapy might be fatal. Furthermore, a previous report showed that the benefits of chemotherapy for de-differentiated liposarcoma are minimal (16). As bee venom is toxic to tumors and also serves as an innate immune system agonist, and because operation, chemotherapy, and radiotherapy were contraindicated, apitherapy combined with camrelizumab was the last resort.

The objective response rates to immune checkpoint inhibitor monotherapies of all malignancies range from 20% to 30% (17, 18). Various factors influence the efficacy of such therapy; all concern immune cell features, particularly the levels and the extents of cooperation among different types of immune cells (19–22). The patient received regular apitherapy because it was clearly analgesic initially. Given that bee venom thus seemed to act as an immune system agonist, we cautiously increased the number of stings. Melittin, which accounts for 50% of venom dry weight, induces G1 cell cycle arrest and apoptosis in ChaGo-K1 human bronchogenic carcinoma cells and inhibits the differentiation of THP-1 cells into tumor-associated macrophages (23). Bee venom enhances the differentiation of human regulatory T cells (24). Melittin-MIL-2 strongly stimulates the immune system and has antitumor effects; the fusion protein promotes IFN-γsecretion in tumor tissues and reduces the levels of immunosuppressive cells *in vivo* (25). These data indicate that bee venom may be a valuable candidate for cancer immunotherapy, given that it activates innate immunity. Activation of the STING-controlled innate immune pathway is a promising therapeutic strategy for cancer, inducing tumor regression with long-term antitumor immunity when synergized with anti-PD-1 therapy. Activation of innate immunity enhances the efficacy of PD-1 inhibitors (26, 27). Bee venom has evolved to become immunotoxic in mammals, as evidenced by the redness and swelling caused by a sting. Such symptoms reflect the rapid activation of pattern recognition receptors including the Toll-like receptor and the nucleotide-binding oligomerization domain-like receptor. Various types of immune cells are then recruited and locally activated. Bee venom per se exhibits strong anti-tumor cytotoxic effects, with negligible side effects (28). It is rich in hyaluronidase, which degrades excess hyaluronan in a tumor microenvironment. This increases penetration of both the venom and other drugs; novel drugs exploiting this principle are in clinical trials (29). The STING pathway is activated by radiation-induced tumor cell apoptosis, DNA double-strand breakage, and micronucleus formation (30, 31). Bee venom induces tumor cell membrane perforation and apoptosis (28, 32), and even lytic death of tumor cells (33). DNA damage, DNA double-stranded breakage (34, 35), and micronucleus formation (36) can be induced by bee venom in a tumor microenvironment. The penetrability afforded by the contained hyaluronidase allows bee venom to penetrate deep into tumor tissues (29). Thus, the venom can activate the STING pathway to change the tumor microenvironment. Compared to radiotherapy and chemotherapy, bee venom is less toxic to normal cells (28) but can induce the recruitment of immune cells and activate immune pathways (37, 38). When bee stings are delivered around a tumor, the venom recruits various types of immune cells that then rapidly infiltrate the tumor microenvironment. At almost the same time, tumor-derived DNA is released into the tumor microenvironment because of the tumor membrane disruption caused by the venom (28), encouraging the infiltration of antigen-presenting cells (APCs) and activation of the STING pathway. Thus, we gradually increased the apitherapy dose (with the patient’s consent), and sought to combine this with a PD-1 inhibitor, although the tumor did not express PD-L1 and no CD8+ infiltrating lymphocytes were detected. The 2017 surgical specimens were subjected to immunohistochemical staining; we used the 22C3 antibody to stain for PD-L1.

Apitherapy triggers local skin hypertrophy and scarring; there are also other potential risks (39, 40). We performed apitherapy in a ward with first-aid equipment at hand and increased the number of bee stings in an orderly and gradual manner. We closely monitored the patient’s vital signs during treatment. Liver, kidney, and heart function were always normal. Daily monitoring revealed slight increases in levels of eosinophils and hyper-sensitive C-reactive protein in peripheral blood. An antibody array revealed that the level of interleukin-1 receptor antagonist in peripheral blood increased gradually; manipulation of this level can by immunotherapeutically useful when seeking to reduce inflammation/immunosuppression and thus enhance anti-tumor immunity (41, 42).

## Conclusions

Given the poor reliabilities of supposedly predictive biomarkers of cancer prognosis, the use of immunotherapies to treat advanced cancers is common when the treatment options are limited, as is the case for advanced liposarcomas. Strategies that enhance the immunotherapeutic effects of such therapies, or extend their scope to other patients, are urgently needed. Herein, we present the case of a 62-year-old male with a chemoradiotherapy-resistant, advanced, de-differentiated liposarcoma. The primary tumor did not express PD-L1, and CD8+ cells were scarce. Preclinically, tumor-derived DNA was evident in the cytosol of tumor-infiltrating APCs after the STING pathway was activated (43). The hyaluronidase of bee venom allows all venom constituents to penetrate deep into tumor tissue; the venom then activates pattern recognition receptors such as the Toll-like receptor and nucleotide-binding oligomerization domain-like receptor. Various types of immune cells are recruited and activated. Bee venom induces tumor cell apoptosis and membrane disruption (28, 44, 45). It thus creates conditions favoring activation of the STING pathway in tumor microenvironments. The STING pathway is a cytosolic DNA-sensing pathway that is considered a promising therapeutic strategy for cancer (46, 47). Downstream STING signaling triggers APC activation and production of inflammatory cytokines, subsequently enhancing T cell priming and recruitment (48). To enhance the therapeutic effect of anti-PD-1 immunotherapy, we rapidly increased apitherapy (an innate immune system agonist) when apatinib was discontinued. Bee venom may effectively activate innate immunity because the venom has anti-tumor cytotoxic effects. It is a biotoxin synthesized and secreted by a gland that is present in the abdominal cavity of the bee and is composed of complex mixture of several biologically active peptides. One drop of bee venom consists of 88% of water and only 0.1 µg of dry venom, and the components of the venom including melittin, adolapin, apamin, and mast cell degranulating peptide. It also contains enzymes, most importantly Phospholipase A2 and hyaluronidase, and compounds of low molecular weight like bioactive amines (e.g., histamine and epinephrine) and minerals (49, 50). Bee venom and many of the contained materials exhibit anti-tumor activities (**Table 1**), the majority of the antineoplastic activity of honeybee venom has been attributed to melittin (57). In recent years, the modification of melittin is mainly focused on reducing the unspecific cytotoxicity of melittin or transforming it into a highly effective immunotherapy drugs (**Table 1**). Our novel immunotherapy regimen requires further in-depth investigation; our present case is but a first step along a new path.

**Table 1:** Selected recent publications about antitumor effects of bee venom and its components or conjugates.

First author and year	Compound	Molecular group	Type of study	cancers	Biological Activity
Duffy C et al. (28)	Pure bee venom & Melittin	Not applicable/Peptide	*In vitro* & *In vivo*	Breast cancer	***In vitro*:** Induce cell death by interfering with growth factor-dependent RTK interactions for receptor phosphorylation and activation of PI3K/Akt signaling; ***In vivo*:** Cell death due to the synergy between melittin and docetaxel
Salama MA et al. (51)	Pure bee venom	Not applicable	*In vitro*	Liver cancer, breast cancer, and cervical cancer	A cytotoxic effect on tumor cells in a dose- and time-dependent manner and regulated caspase independent pathway inducing apoptosis; Cell death due to the synergy between plasma-treated phosphate buffered saline solution and melittin
Yu X et al. (52)	α-melittin-NPs	Modified peptide (nanovaccine)	*In vivo*	Melanoma	Promotes whole tumor antigen release *in situ* and results in the activation of antigen-presenting cells, thus primary and distant tumor growth are inhibited
Yu X et al. (37)	Melittin nanoparticles	Modified peptide	*In vivo*	Melanoma, breast cancer, and colon cancer	Melittin nanoparticles trigger the activation of liver sinusoidal endothelial cells and lead to dramatic changes of cytokine/chemokine milieu in the liver, which switches the hepatic immunologic environment to the activated state to inhibit liver metastasis
Shaw P et al. (53)	Melittin	Peptide	*In vitro*	Melanoma, and breast cancer	Cell death due to the synergy between plasma-treated phosphate buffered saline solution and melittin, meanwhile it help to reduce the non-specific toxicity of melittin
Jung GB et al. (54)	Pure bee venom	Not applicable	*In vitro*	Breast cancer	Denaturation and degradation of proteins and DNA fragmentation associated with cell death
Luo L et al. (44)	MLT-DMMA	Modified peptide	*In vitro*	Cervical cancer	A steady cytotoxic effect on tumor cells, with the ability to shield hemolytic and the unspecific cytotoxicity
Su MM et al. (55)	ATF-melittin	Modified peptide	*In vitro*	Ovarian cancer	Inhibited growth of cancer cells with no cytotoxicity on normal cells
Liu MJ et al. (25)	Melittin-MIL-2 fusion protein	Modified peptide	*In vivo*	Breast cancer	Inducing T cell and NK-cell cytotoxicity; Inhibited the growth of tumors **In vivo* via* increasing of IFN-γ production in PBMCs; Decreasing the immunosuppressive cells causing reduced lung metastasis of breast cancer
Shao GC et al. (56)	EGFP- M-IL-2	Modified peptide	*In vitro*	Cervical cancer	Inhibited cell proliferation and induced apoptosis in the tumor cells

## Data Availability Statement

The original contributions presented in the study are included in the article/supplementary material. Further inquiries can be directed to the corresponding author.

## Ethics Statement

The studies involving human participants were reviewed and approved by institutional research ethics committee of Hangzhou Red Cross Hospital. The patients/participants provided their written informed consent to participate in this study. Written informed consent was obtained from the individual(s) for the publication of any potentially identifiable images or data included in this article.

## Author Contributions

WY, DX, FH, and XX designed, organized, and supervised the study. WY, GY, YY, and HW drafted the manuscript. WY, JW, and DC analyzed the literature. DX and XX revised the manuscript. YZ, YG, JJ, and FH participated in the revision. All authors contributed to the article and approved the submitted version.

## Funding

The Medical and Health Research Project Funded by the Hangzhou Municipal Health and Family Planning Commission (Project Number: 0020190125, 0020190293 and ZD20200004).

## Conflict of Interest

The authors declare that the research was conducted in the absence of any commercial or financial relationships that could be construed as a potential conflict of interest.
